# Antimicrobial resistance and virulence profiles of *Enterobacterales* isolated from two-finger and three-finger sloths (*Choloepus hoffmanni* and *Bradypus variegatus*) of Costa Rica

**DOI:** 10.7717/peerj.12911

**Published:** 2022-03-11

**Authors:** Matilde Fernandes, Carla Nóbrega Carneiro, Ana Maria Villada Rosales, Miguel Grilo, Yolanda Ramiro, Eva Cunha, Telmo Nunes, Luís Tavares, Janet Sandi, Manuela Oliveira

**Affiliations:** 1 CIISA–Centro de Investigação Interdisciplinar em Sanidade Animal, Faculdade de Medicina Veterinária, Universidade de Lisboa, Lisboa, Lisboa, Portugal; 2 Toucan Rescue Ranch (TRR), Heredia, San Josecito, Costa Rica

**Keywords:** Antimicrobial resistance, Bacterial virulence factors, Wildlife bacteria, One health, *Choloepus hoffmanni*, *Bradypus variegatus*

## Abstract

**Background:**

Wildlife has been recently recognized as an environmental reservoir for antimicrobial resistance (AMR). However, less information on this topic is available in animals released back into the wild after rehabilitation in wildlife facilities, compared with studies performed exclusively in captive or free-ranging wildlife. This study aimed to evaluate the potential influence of captivity and/or treatment while in captivity of wild sloths on the AMR and virulence profiles of sloths’ *Enterobacterales*.

**Methods:**

Oral and rectal swab samples were collected from 39 two-finger (*Choloepus hoffmanni*) and three-finger sloths (*Bradypus variegatus*) of Costa Rica (*n* = 78) and analyzed using conventional bacteriological techniques. A generalized linear mixed model was applied to estimate the isolates’ multiple antimicrobial resistance and virulence indices as a function of animal status.

**Results:**

A considerable level of resistance was detected, especially for *Citrobacter youngae* and *Escherichia coli*, with 17.5% of isolates classified as multidrug-resistant. Virulence indices of isolates from rehabilitated sloths were significantly higher than the ones from sloths being hand-reared for shorter periods.

**Conclusions:**

To our knowledge, this is the first description of sloths’ antimicrobial resistant *Enterobacterales*, suggesting that sloths’ rehabilitation and consequent exposure to humans, may promote the selection of bacteria with higher virulence. Ultimately, these bacteria may represent a threat to human and animal health due to their zoonotic potential and AMR and virulence profiles.

## Introduction

Antimicrobial resistance (AMR) is a major threat to human and animal health worldwide, impairing the capacity to treat important infections (Vittecoq et al., 2016).

Considering the increasing concern on AMR, recent studies highlighted the importance of recognizing the role of different animal populations (*e.g*., human populations, domestic animals, and wildlife) in the persistence and dissemination of multiple antimicrobial-resistant (AMR) strains, particularly those with a virulent profile (Radhouani et al., 2014; Vittecoq et al., 2016). Despite the high level of attention being raised to the AMR dynamics in humans’ populations and domestic animals, studies focusing on wildlife still fall behind (Radhouani et al., 2014). Nevertheless, the role played by wildlife in the dissemination of antimicrobial-resistant bacteria (ARB) harboring virulence factors is increasingly acknowledged (Da Costa, Loureiro & Matos, 2013; Fernandes et al., 2021). Furthermore, there is increasing evidence that wildlife populations in proximity to anthropogenic-dominated habitats harbor higher levels of ARB compared with wildlife living in pristine environments, which supports the hypothesis that most AMR detected in wild environments might be shaped by human activities (Power, Emery & Gillings, 2013). A recent study on antimicrobial resistance genes (ARGs) detected in fecal samples of wild felids from Chile revealed a statistically significant difference on the level of AMR between animals from anthropized and pristine areas. This study showed that animals from anthropized areas harbored higher levels of multidrug resistance (Sacristán et al., 2020). Also, significantly higher levels of antimicrobial-resistant *Escherichia coli* were detected in elephant seals (*Mirounga angustirostris*) after rehabilitation compared to the levels detected at animals’ admission (Stoddard et al., 2009). Moreover, mobile genetic elements (class 1 integrons) associated with clinical resistance in Gram-negative bacteria were detected in captive brush-tail rock wallabies (*Petrogale penicillata*), while no integrons were detected in wild populations of this species (Power, Emery & Gillings, 2013).

Virulence factors are described as inherent elements of a pathogen with the ability to cause damage to host cells and tissues, as well as molecules or structures (*e.g*., biofilm) that enable the pathogen to evade or modulate host defense mechanisms to its replicative advantage (Johnson, 2017). The expression of a high number of virulence factors may play an essential role in the pathogenesis of infections (Johnson, 2017). Pavlickova et al. (2017) detected several virulence genes in *E. coli* isolates from wildlife (pheasants and mallards), especially the ability to form biofilms (60% positive strains); Semedo-Lemsaddek et al. (2013), identified different enterococci species isolated from wild Eurasian otters (*Lutra lutra*), with the ability to produce virulence factors, including gelatinases. Finally, Fernandes et al. (2021), revealed that bacteria isolated from wild loggerhead turtles were able to produce several virulence factors, including biofilms, hemolysins, DNases, and lipases.

Despite this evidence, a thorough understanding of the extent and dynamics of virulence and AMR in wildlife bacteria is far from being accomplished.

Sloths are arboreal mammals from the Xenarthra superorder. Two-finger (*Choloepus hoffmanni*) and three-finger sloths (*Bradypus variegatus*) live in the canopy of the Neotropics, being exclusively found in Central and South America, especially in Costa Rica (Dill-Mcfarland et al., 2016). Two-finger sloths (*C. hoffmanni*) are characterized by having two fingers and three toes, large canines, and an omnivorous diet. *B. variegatus* presents three fingers and three toes, are heterothermic and strict folivores (Dünner & Pastor, 2017). Although *Choloepus hoffmanni* and *Bradypus variegatus* are classified as ‘least concern’ under the IUCN (International Union for the Conservation of Nature) criteria, information on population size, distribution, and demographic trends is scarce (Moraes-Barros, Chiarello & Plese, 2014; Plese & Chiarello, 2014). In Costa Rica, the territory covered by an adult sloth is approximately two hectares, considering that ranges can be smaller for three-finger sloths and females (M Fernandes, 2021, personal communication). The territory range depends mainly on the availability of food, which has decreased in the past years due to the destruction of sloths’ habitats, forcing these animals to travel greater distances. The disturbance of sloths’ natural habitats also exposes them to several risks, including electrocutions by unprotected powerlines and attacks by domestic dogs, which affect both adult animals and infants that might lose their mothers in such traumatic events (M Fernandes, 2021, personal communication). In fact, these are two main reasons for the growing numbers of sloths admitted to wildlife facilities, which require intensive medical care or hand-rearing by wildlife professionals (M Fernandes, 2021, personal communication). Ultimately, these procedures establish an uncommon interface between humans and wildlife, which may promote the dissemination of bacteria between these host groups as well as the exchange of AMR and virulence factors (Power, Emery & Gillings, 2013).

In this study, we assessed the role of two-finger and three-finger sloths as carriers and potential spreaders of *Enterobacterales* species with a resistance profile relevant to human and veterinary medicine. We also aimed to assess the pathogenic potential of these bacterial species by determining their ability to express different virulence factors. Finally, we evaluated the influence of hand-rearing and rehabilitation of wild sloths, which implies exposure to human environments, on the antimicrobial resistance and virulence profiles of sloths’ *Enterobacterales*.

Our findings revealed that rehabilitated sloths can carry *Enterobacterales* species with AMR and virulence profiles relevant to both human and veterinary medicine, namely resistances towards critically important antimicrobial classes, such as carbapenems (meropenem), fluoroquinolones (enrofloxacin) and cephalosporins (ceftazidime) (McEwen & Collignon, 2018). Ultimately, these results suggest that released sloths may play an important role in the dissemination of AMR.

## Materials and Methods

### Sample collection

#### Area of study

The area of the study comprised the Headquarters, San Jose (Lat: 10°57′6.23″N; Long.: −85°08′9.96″W) and Release Site, Sarapiqui (Lat: 10°28′24.6828″N; Long: −84°1′0.2712″W) of the wildlife facility “Toucan Rescue Ranch” (TRR), in Costa Rica (Lat: 9°37′48.68″N; Long: −84°15′15.06″W) (Fig. S1). San Josecito is a populated city located in the Heredia province, only 16.8 km north of San Jose, the country capital. San Jose is a highly urbanized city, concentrating large industrial centers. On the other hand, TRR’s release site (Sarapiqui), is mostly characterized by a natural environment with a high density of wildlife. Sarapiqui is also place of some human activity, as well as domestic and farm animals.

#### Animals

Two-finger (*Choloepus hoffmanni*) and three-finger sloths (*Bradypus variegatus*), from the Headquarters and Release Site of the wildlife facility “Toucan Rescue Ranch”, were selected for sampling. Animals were categorized into three different status groups: ‘hand-reared’, ‘rehabilitated and released’, and ‘wild’ sloths. Orphaned infant or new-born sloths in need to be hand-reared by wildlife professionals were classified as ‘hand-reared’. Sloths in pre-released enclosures or released in their natural habitat but tracked with collars after being treated and rehabilitated at TRR were classified as ‘rehabilitated and released’. Wild sloths captured in nature for a general health evaluation or at admission to the clinic (*e.g*., orphaned or injured) were categorized as ‘wild’. Healthy wild animals captured in the wild were immediately released after examination and sampling.

At the time of sampling, the time spent at TRR, and the length of antimicrobial treatment differed for the different groups. ‘Hand-reared’ sloths were kept in captivity for an average of 6 months (from 1 to 12 months) (Table S2) and antimicrobial treatment did not exceed 7 days. On the other hand, the average time of rehabilitation for ‘rehabilitated and released’ animals, was 1.9 years (from 1 to 3 years) (Table S2) and the length of antimicrobial treatment was approximately 3 weeks. The antimicrobials mostly used for the sloths under study were enrofloxacin, amikacin, and amoxicillin-clavulanate (Table S2). Previous diseases are described in Table S2. ‘Wild’ sloths were sampled at admission or general health checks and did not receive antimicrobial treatment at the time of sampling (Table S2). Rehabilitated and released sloths were tracked with collars and sampled between 2 months and 2 years after release. By the time of sampling, these sloths covered an area of approximately seven hectares.

#### Sampling procedures

AMIES swab samples (VWR, Leuven, Belgium) were collected from the sloths, between November 2019 and March 2020, at TRR’s Headquarters and Release Site in Costa Rica. Oral and rectal swab samples were collected from each animal during general health checks upon admission at the clinic, during rehabilitation or on periodical general checks of released animals with tracking collars.

Oral samples were collected by gently opening the mouth of the animal and swabbing the tongue and palate, rolling the swab for approximately 5 s. Rectal samples were collected by inserting a swab approximately 2 cm into the rectum and, with a rotative movement, collecting the fecal material present in the rectal dilation. The samples were kept at 4 °C until transportation to the Microbiology and Immunology Laboratory from the Veterinary Faculty in Lisbon, Portugal, for further processing. In all procedures, the animals were adequately restrained. The handling time did not exceed 5 min per animal, and stress was minimized by providing a safe and calm environment. Personal protective equipment was used.

The following information was collected regarding each sample: date of sampling, place of sampling (Headquarters or Release Site), identification of the animal, type of sample (oral or rectal), animal status (‘hand-reared’, ‘rehabilitated and released’, ‘wild’), reason for admission (*e.g*., electrocution), clinical history (*e.g*., pneumonia), previous antimicrobial treatment, time of sampling (*e.g*., 2 months after admission), and time in captivity (Table S2).

### Isolation of *Enterobacterales*

After pre-enrichment in Buffered Peptone Water broth (VWR, Leuven, Belgium) at 37 °C for 24 h, Gram-negative aerobic bacteria were isolated from collected samples using MacConkey Agar (Oxoid, Hampshire, UK), incubated at 37 °C for 24 h. Pink colonies (lactose-positive) surrounded by a turbid zone due to the precipitation of bile acids were isolated in Columbia Agar with 5% sheep blood (BioMérieux, Marcy-l’Etoile, France), incubated at 37 °C for 24 h for identification confirmation (Alduina et al., 2020).

Pre-enrichment suspensions in Buffered Peptone Water broth were also inoculated in Rappaport Vassiliadis broth (Biokar Diagnostics, Beauvais, France), followed by incubation at 37 °C for 24 h. Bacterial suspensions in Rappaport Vassiliadis broth were inoculated onto Hektoen Enteric Agar (Oxoid, Hampshire, UK) and Brilliant Green Agar (Liofilchem, Teramo, Italy), incubated at 37 °C for 24 h (Alduina et al., 2020). Black colonies with a transparent halo (Hektoen Enteric Agar) and pink colonies (Brilliant Green Agar) were selected and inoculated in Triple Sugar Iron Agar (TSI) (Scharlau, Barcelona, Spain), at 37 °C for 24 h. TSI colonies revealing one of the following profiles: yellow slant + yellow butt + gas; red slant + yellow butt; red slant + yellow butt + H_2_S (sulfuric acid)-production (blackening of the medium); red slant + H_2_S-production, were selected and isolated in Columbia Agar with 5% sheep blood, at 37 °C for 24 h, for further identification (Midorikawa et al., 2014).

Macro and microscopic morphology, Gram staining, and enzymatic reactions (*e.g*., catalase and oxidase) were assessed for all isolates.

### Identification of the isolates

The IMVic test (indole, methyl red, Voges-Proskauer, and citrate) was used for *Escherichia coli* identification (Powers & Latt, 1977). Gram-negative, lactose-positive, oxidase negative colonies isolated from MacConkey Agar were inoculated in three culture media: Simmons Citrate Agar (Oxoid, Hampshire, UK), SIM Medium (Merck, Darmstadt, Germany) and MR-VP Broth (Oxoid, Hampshire, UK). The Simmons Citrate Agar was used to test the ability to utilize citrate as a carbon source, while the SIM Medium was used to test motility and sulphide and indole production. Citrate negative species (inhibited growth in Simmons Citrate Agar), motile (diffuse evened growth and turbidity of the SIM medium), non-hydrogen sulphide producers (absence of blackening of the SIM medium) and indole producers (immediate change of the reagent layer color from encolour to pink after addition of James reagent) were identified as *Escherichia coli*. On the Methyl Red-Voges-Proskauer (MR-VP) test, *E. coli* isolates did not promote the change of color of the medium following the addition of the reagents VP1 and VP2. The reference strain *Escherichia coli* ATCC^®^ 25922™ was used for quality control (Powers & Latt, 1977; Visser, Jaisly & Mossel, 1985).

Gram-negative, oxidase negative bacilli isolated from TSI Medium were identified by the biochemical gallery API 20E (BioMérieux, Marcy-l’Etoile, France), accordingly to the manufactures’ instructions.

### Evaluation of isolates’ antimicrobial resistance profile

Isolates’ susceptibility profile was determined using the disk diffusion method, according to the Clinical and Laboratory Standards Institute (CLSI) guidelines and following established breakpoints (CLSI, 2020). Briefly, isolates were cultured in Columbia agar supplemented with 5% sheep blood (BioMérieux, Marcy-l’Etoile, France) and incubated at 37 °C for 24 h. Afterwards, a 10^8^ CFU/mL bacterial suspension, with a 0.5 turbidity in the McFarland scale, was prepared in a sterile saline solution. The bacterial suspension was evenly spread over a Mueller-Hinton agar (VWR, Leuven, Belgium) plate, and antibiotic-impregnated disks were placed over the surface of the agar plates, which were incubated at 37 °C for 18 h.

We selected twelve different antimicrobials belonging to six different classes, and commonly used in veterinary and human medicine. The tested antimicrobials (Oxoid, Hampshire, UK and Mast Group, Bootle, UK), included: aminoglycosides (gentamicin (GEN, 120 µg), amikacin (AMK, 30 µg)); cephalosporins (cefalexin (CFX, 30 µg), ceftazidime (CAZ, 30 µg)); fluoroquinolones (ciprofloxacin (CIP, 5 µg), enrofloxacin (ENR, 5 µg)); carbapenems (imipenem (IMP, 10 µg), meropenem (MEM, 10 µg)); tetracycline (tetracycline (TET, 30 µg)); β-Lactams (amoxicillin-clavulanate (AMC, 30 µg)); penicillins (ampicillin (AMP, 10 µg)); phenicols (chloramphenicol (CAP, 30 µg)). The CLSI guidelines were used to classify the inhibition zones as susceptible, intermediate, or resistant (CLSI, 2020), using the reference strain *Escherichia coli* ATCC^®^ 25922™ as quality control. Intrinsic resistances were considered, following Magiorakos et al., 2012 classification. A 10% replica was performed in independent days, by repeating the antimicrobial susceptibility testing of 10% randomly selected isolates (CLSI, 2020). The antimicrobial compounds were selected based on their use in human and veterinary medicine, including compounds commonly used in Costa Rica’s wildlife facilities (*e.g*., enrofloxacin, amoxicillin-clavulanate, amikacin) (M Fernandes, 2021, personal communication).

### Evaluation of isolates’ virulence profile

The phenotypic virulence profile was assessed for all isolates, by evaluating the ability of the isolates to produce enzymes associated with bacterial pathogenicity.

Hemolysins’ production was analyzed as described by Fernandes et al. (2021), using Columbia Agar with 5% sheep blood (BioMérieux, Marcy-l’Etoile, France) (Fernandes et al., 2021).

DNase activity was tested using DNase Agar supplemented with 0.005% methyl green (VWR, Leuven, Belgium), using *Aeromonas hydrophila* ATCC^®^ 7966™ and *Escherichia coli* ATCC^®^ 25922™ as positive and negative controls, respectively (Elder, Trujillo & Blazevic, 1977).

Lipase activity was evaluated using Spirit Blue agar (Difco, Detroit, USA) supplemented with 0.25% Tween^®^ 80 (AppliChem GmbII, Darmstadt, Germany) and 25% olive oil (commercial), using *Pseudomonas aeruginosa* ATCC^®^ 27853™ and *Staphylococcus aureus* ATCC^®^ 29213™ as positive and negative controls, respectively (Fernandes et al., 2021).

Lecithinase activity was determined using Tryptic Soy Agar (VWR, Leuven, Belgium) combined with 10% egg yolk emulsion (VWR, Leuven, Belgium). *Pseudomonas aeruginosa* ATCC^®^ 27853™ and *Escherichia coli* ATCC^®^ 25922™ were used as positive and negative controls, respectively (Chrisope, Fox & Marshall, 1976).

Protease activity was assessed by resorting to Skim Milk powder (Oxoid, Hampshire, UK) supplemented with Bacteriological Agar (VWR, Leuven, Belgium). *Pseudomonas aeruginosa* ATCC^®^ 27853™ and *Staphylococcus aureus* ATCC^®^ 29213™ were used as positive and negative controls, respectively (Sokol, Ohman & Iglewski, 1979).

Gelatinase activity was detected resorting to Nutrient Gelatin Agar (Oxoid, Hampshire, UK), using *Pseudomonas aeruginosa* ATCC^®^ 27853™ and *Escherichia coli* ATCC^®^ 25922™ as positive and negative controls, respectively (Fernandes et al., 2021).

The ability of the tested isolates to produce biofilms was assessed using Congo Red Agar Plates, composed of Brain Heart Infusion broth (VWR, Leuven, Belgium), Bacteriological Agar (VWR, Leuven, Belgium) and 0.0008% Congo Red indicator (Sigma Aldrich, St. Louis, USA). *Enterococcus faecium* ATCC^®^ 35667™ and *Escherichia coli* ATCC^®^ 25922™ were used as positive and negative controls, respectively (Freeman, Falkiner & Keane, 1989).

For the testing of all virulence factors, the plates were incubated at 37° C for 24 h.

### Statistical analysis

The multiple antimicrobial resistance index (MAR index) (Eq. (1)) and the virulence index (V. Index) (Eq. (2)) values were determined for all isolates (Krumperman, 1983; Singh et al., 2017).

(1) $$MAR\; index = \displaystyle{{no.\; \; antimicrobials\; to\; which\; isolates\; were\; resistant} \over {no.\; \; antimicrobials\; tested}}$$

(2) $$V.\; index = \displaystyle{{no.\; \; positive\; virulence\; factors} \over {no.\; \; virulence\; factors\; tested}}$$

A generalized linear mixed model (GLMM) was performed to estimate the isolates’ multiple antimicrobial resistance and virulence indices as a function of animal status (‘hand-reared’, ‘rehabilitated and released’ and ‘wild’), sex (‘male’, ‘female’), previous antimicrobial treatment, and time in captivity (‘more than 1 year’, ‘less than one year’). For this evaluation, we selected the Rpackage lme4 v.1.1.26 (Bates et al., 2015). The model included V. Index, MAR index, Animals Status, Sex, Antimicrobial Treatment, and Time in Captivity as fixed effects, with the variable Animal (sloth identification) used as a random effect. The factor Species was not included in the model, due to the low number of samples collected from three-finger sloths (*B. variegatus*) (*n* = 8).

A GLMM was selected as it allows to model correlated, nonnormally distributed data. A Holm-Bonferroni correction on the *p* values was performed to correct for multiple comparisons (Holm, 1979). The significance level (*p*) was set to 0.05 for the statistical tests. All statistical analysis were performed in R-Studio (Version; R version 4.0.3). Bacterial species, antimicrobial resistance, and virulence reported as frequencies, MAR index and V. Index mean values, and the percentages of resistance for different antimicrobials were calculated in Microsoft Excel for Microsoft 365 MSO.

### Ethics statement

The present study was performed in accordance with the rules established by the current EU (Directive 2010/63/EC) and national (DL 113/2013) legislation and by the competent authority (Direção Geral de Alimentação e Veterinária, DGAV, www.dgv.min-agricultura.pt/portal/page/portal/DGV) in Portugal. The samples of this study were collected by trained veterinary surgeons, during standard routine procedures and following Toucan Rescue Ranch (U.S. 501(c)3 Non-Profit) guidelines. This study did not include any animal experiment, and only non-invasive samples were collected during sampling procedures.

## Results

A total of 78 samples were collected from 39 animals, including one rectal and one oral swab sample per animal. Two-finger (*n* = 35) and three-finger sloths (n = 4) were grouped in three different categories regarding the status of the animal: ‘hand-reared’ (*n* = 16; 41.0%), ‘rehabilitated and released’ (*n* = 17; 43.6%), and ‘wild’ (*n* = 6; 15.3%).

From the collected samples, it was possible to obtain 13 isolates from oral samples (32.5%) and 27 isolates from rectal samples (67.5%), making a total of 40 isolates (Table 1).

**Table 1:** Frequency of isolates from the identified bacterial species isolated from different sample types and animal status groups.

Bacterial species	Frequency of isolates (*n* = x) (%)	Sample type		Animal status		
		Oral	Rectal	Hand-reared	Rehabilitated and released	Wild
*E. coli*	22 (55.0)	1 (2.5)	21 (52.5)	13 (32.5)	9 (22.5)	0 (0.0)
*C. youngae*	9 (22.5)	5 (12.5)	4 (10.0)	1 (2.5)	8 (20.0)	0 (0.0)
*E. amnigenus*	3 (7.5)	3 (7.5)	0 (0.0)	2 (5.0)	1 (2.5)	0 (0.0)
*Salmonella* spp.	2 (5.0)	1 (2.5)	1 (2.5)	0 (0.0)	1 (2.5)	1 (2.5)
*E. cloacae*	2 (5.0)	2 (5.0)	0 (0.0)	1 (2.5)	1 (2.5)	0 (0.0)
*Shigella* sp.	1 (2.5)	1 (2.5)	0 (0.0)	1 (2.5)	0 (0.0)	0 (0.0)
*E. aerogenes*	1 (2.5)	0 (0.0)	1 (2.5)	0 (0.0)	0 (0.0)	1 (2.5)
Total (*n* = x) (%)	40 (100.0)	13 (32.5)	27 (67.5)	18 (45.0)	20 (50.0)	2 (5.0)

**Note:**

Number of isolates (*n* = x), percentage (%).

Not all animals were positive for *Enterobacterales* species, with some animals being negative for these bacteria and other positive for two different species (Tables S1, S2). Isolates were identified as *Escherichia coli* (*n* = 22; 55.0%), *Citrobacter youngae* (*n* = 9; 22.5%), *Enterobacter amnigenus* (*n* = 3; 7.5%), *Salmonella* spp. (*n* = 2; 5.0%), *Enterobacter cloacae* (*n* = 2; 5.0%), *Shigella* sp. (*n* = 1; 2.5%), and *Enterobacter aerogenes* (*n* = 1; 2.5%) (Table 1).

*Shigella* sp., *E. cloacae* and *E. amnigenus* were exclusively isolated from oral samples. *E. aerogenes* and all except one *E. coli* isolates were recovered from rectal swab samples (Table 1).

The frequency of isolates from each animal status groups was as follows: ‘hand-reared’ (45.0%; *n* = 18), ‘rehabilitated and released’ (50.0%; *n* = 20) and ‘wild’ (5.0 %; *n* = 2) (Table 1).

### Characterization of isolates’ antimicrobial resistance profile

Isolates presented the highest levels of resistance towards meropenem (55.0%), followed by amoxicillin/clavulanate (40.0%), and cephalexin (27.5%). All isolates were susceptible to gentamicin (100.0%), presenting the lowest levels of resistance for the aminoglycoside class (Table 2).

**Table 2:** Antimicrobial resistance profiles of *Enterobacterales* isolates obtained from sloths.

Antimicrobial class	Antimicrobial compound (dose)	Bacterial isolates (*n* = x) (%)		
		Susceptible	Intermediate	Resistant
β-Lactam Combination agents	Amoxicillin/clavulanate (10 µg)	19 (47.5)	3 (7.5)	16 (40.0)
Penicillins	Ampicillin (10 µg)	36 (90.0)	2 (5.0)	2 (5.0)
Carbapenems	Imipenem (10 µg)	39 (97.5)	1 (2.5)	0 (0.0)
	Meropenem (10 µg)	15 (37.5)	3 (7.5)	22 (55.0)
Fluoroquinolones	Ciprofloxacin (5 µg)	38 (95.0)	0 (0.0)	2 (5.0)
	Enrofloxacin (5 µg)	33 (82.5)	3 (7.5)	5 (12.5)
Aminoglycosides	Amikacin (30 µg)	39 (97.5)	0 (0.0)	1 (2.5)
	Gentamicin (120 µg)	40 (100.0)	0 (0.0)	0 (0.0)
Cephalosporins	Cephalexin (30 µg)	19 (47.5)	10 (25.0)	11 (27.5)
	Ceftazidime (30 µg)	35 (87.5)	3 (7.5)	2 (5.0)
Tetracyclines	Tetracycline (30 µg)	35 (87.5)	1 (2.5)	4 (10.0)
Phenicols	Chloramphenicol (30 µg)	36 (90.0)	2 (5.0)	2 (5.0)

**Note:**

Number of isolates (*n* = x), percentage (%), micrograms (µg).

The prevalence of bacteria non-susceptible to three or more antimicrobials was 40.0%. According to Magiorakos et al., 2012 classification, 17.5% of isolates (*E. coli*—10.0% (*n* = 4) and *C. youngae*—7.5% (*n* = 3)) were categorized as multidrug-resistant (MDR) since they exhibited non-susceptibility to at least one antimicrobial agent in three or more antimicrobial classes. One *E. coli* isolate was defined as extensively drug-resistant (XDR), as it presented non-susceptibility to one or more agents in all except two of the tested antimicrobial categories (Table S1) (Magiorakos et al., 2012).

**Figure 1:**
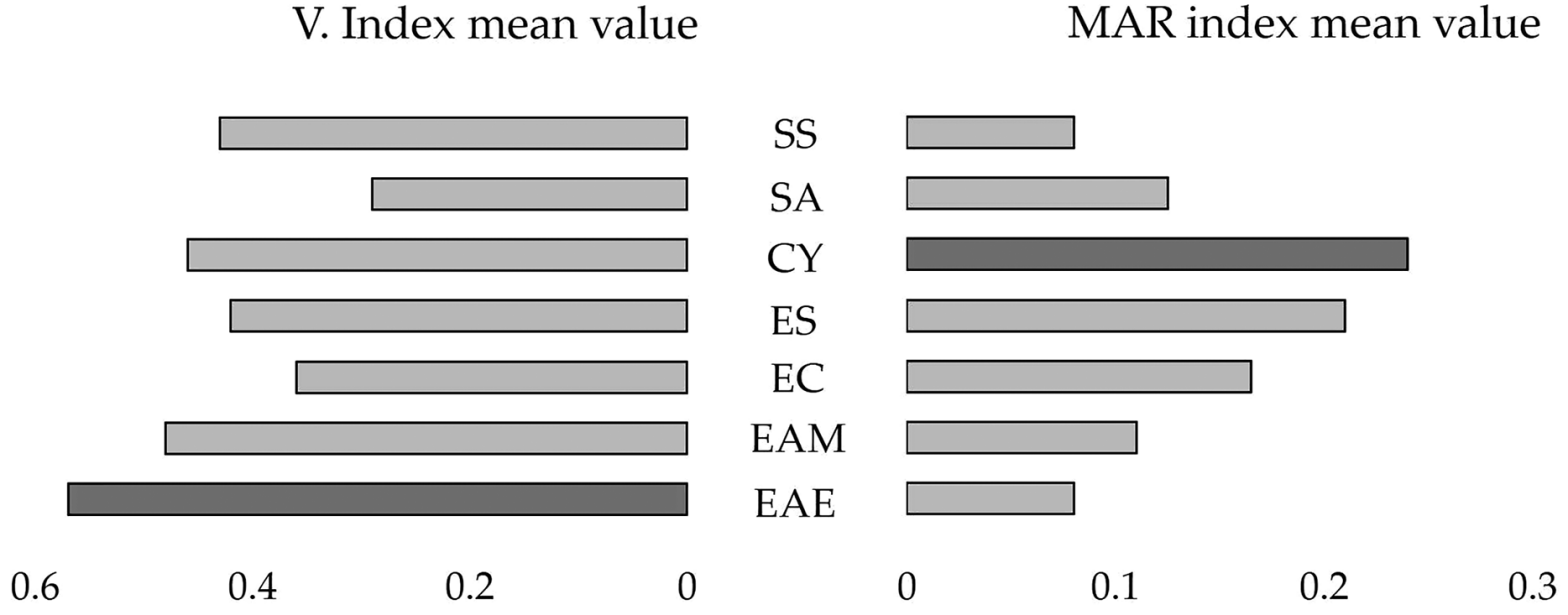
Mirror bar chart of the Virulence Index (V. Index) and MAR index mean values for the isolates of the identified bacterial species. *Shigella sonnei* (SS), *Salmonella enterica* subspecies *arizonae* (SA), *Citrobacter youngae* (CY), *Escherichia coli* (ESC), *Enterobacter cloacae* (EC), *Enterobacter amnigenus* (EAM), *Enterobacter aerogenes* (EAE). The bacterial species with the highest V. Index and MAR index mean values are highlighted in dark grey.

The highest MAR index mean values were detected for *C. youngae* (MAR index mean value = 0.24) and *E. coli* (MAR index mean value = 0.21) (Fig. 1).

**Figure 2:**
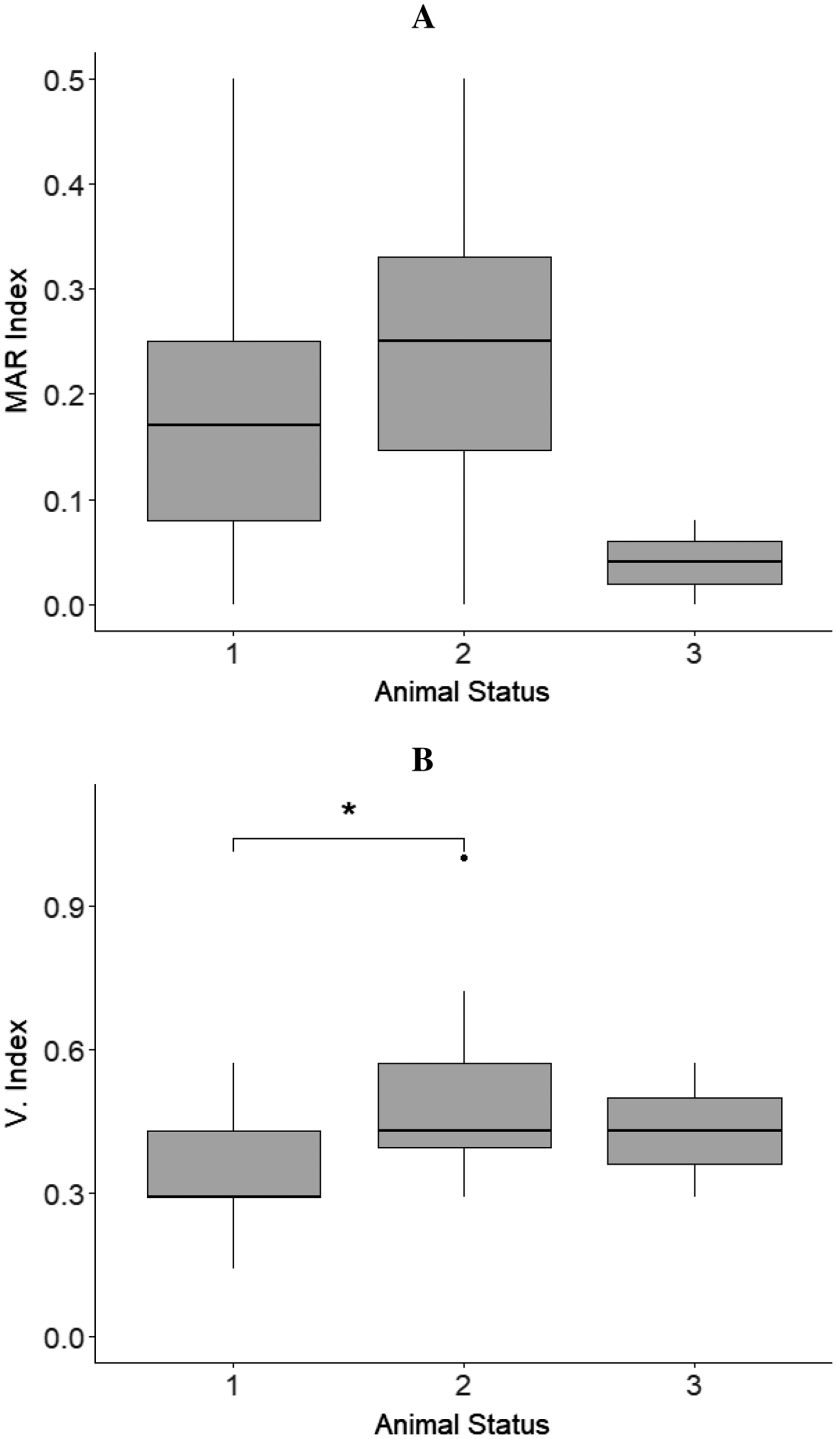
Results from boxplots analysis for MAR and Virulence index (V. Index) for isolates from sloths of each animal status group. (A) MAR index and (B) V. index mean values calculated for the isolates obtained from sloths of the different animal status groups: ‘hand-reared’ (1), ‘rehabilitated and released’ (2), and ‘wild’ (3). The sensitivity level between V. Index mean values of isolates from samples of ‘hand-reared’ (1) and ‘rehabilitated and released’ (2) sloths is indicated by * *p* < 0.05. The boxplots were created using Rpackage “ggplot2” (Wickham, 2016).

The MAR index mean values of isolates from sloths belonging to different animal status groups were as follows: hand reared‒MAR index mean value = 0.18, rehabilitated and released‒MAR index mean value = 0.22, and wild‒MAR index mean value = 0.04 (Fig. 2A).

No statistically significant differences were observed for the isolates’ MAR indices between animals belonging to the different status groups, sloths that had or had not received antimicrobial treatment (*p* = 0.369), female and male sloths (*p* = 0.493), and sloths that where in captivity for less or more than one year (*p* = 0.369) (Table 3).

**Table 3:** Generalised linear mixed models (GLMM) used for the analysis of the multiple antibiotic resistance (marindex) and virulence index (virindex) data.

Model	Predictor	β	SE	*df*	*t-value*	*p*	*p*_adj_
virindex ~ animalstatus + (1|animal)	Hand-reared : Released	0.139	0.052	36.000	2.658	0.012	0.016
	Hand-reared : Wild	0.075	0.119	36.000	0.634	0.530	–
marindex ~ animalstatus + (1|animal)	Hand-reared : Released	0.044	0.048	20.194	0.922	0.367	–
	Hand-reared : Wild	−0.144	0.100	32.697	−1.433	0.161	–
marindex ~ antibiotictx + (1|animal)	No antibiotic treatment : Antibiotic treatment	0.044	0.048	21.052	0.917	0.369	–
marindex ~ sex + (1|animal)	Female : Male	−0.034	0.048	21.496	−0.698	0.493	–
virindex ~ sex + (1|animal)	Female : Male	0.037	0.059	21.334	0.618	0.543	–
marindex ~ time in captivity	≤1 year : ≥1 year	0.045	0.048	20.819	0.921	0.368	–
virindex ~ time in captivity	≤1 year : ≥1 year	0.133	0.051	37.000	2.627	0.013	0.023

**Note:**

The intercept (β), standard error (SE), degrees of freedom (df), test statistics (t-value), *P*-value and Holm-Bonferroni-adjusted *P*-value (*p*_adj_) (if appropriate) are presented for the predictors in each model (reference intercept specified with “:”).

### Characterization of isolates’ virulence profile

The tested isolates were able to produce lipases (100.0%), biofilms (60.0%), hemolysins (55.0%), proteases (50.0%) and DNases (32.5%). Gelatinase (2.5%) and lecithinase (2.5%) activities were less detected for the isolates under study (Table 4).

**Table 4:** Virulence profiles of *Enterobacterales* isolates obtained from sloths.

Bacterial species	Virulence profile (*n* = x) (%)						
	HEM	DNase	GEL	LEC	PT	LIP	BF
*E. coli*	7 (17.5)	7 (17.5)	1 (2.5)	1 (2.5)	12 (30.0)	22 (55.0)	15 (37.5)
*E. cloacae*	2 (5.0)	0 (0.0)	0 (0.0)	0 (0.0)	1 (2.5)	2 (5.0)	0 (0.0)
*E. amnigenus*	2 (5.0)	1 (2.5)	0 (0.0)	0 (0.0)	2 (5.0)	3 (7.5)	2 (5.0)
*E. aerogenes*	1 (2.5)	0 (0.0)	0 (0.0)	0 (0.0)	1 (2.5)	1 (2.5)	1 (2.5)
*C. youngae*	8 (20.0)	4 (10.0)	0 (0.0)	0 (0.0)	4 (10.0)	9 (22.5)	5 (12.5)
*Shigella* sp.	1 (2.5)	0 (0.0)	0 (0.0)	0 (0.0)	0 (0.0)	1 (2.5)	1 (2.5)
*Salmonella* spp.	1 (2.5)	1 (2.5)	0 (0.0)	0 (0.0)	0 (0.0)	2 (5.0)	0 (0.0)
Total (*n* = x) (%)	22 (55.0)	13 (32.5)	1 (2.5)	1 (2.5)	20 (50.0)	40 (100.0)	24 (60.0)

**Note:**

HEM, Hemolysins; DNase, DNases; GEL, gelatinases; LEC, lecithinases; PT, proteases; LIP, lipases; BF, biofilms; number of isolates (*n* = x), percentage (%).

The highest virulence indices were detected for the isolates identified as *E. aerogenes* (V. Index mean value = 0.57) and *E. amnigenus* (V. Index mean value = 0.48) (Fig. 1). One *E. coli* isolate was able to produce all virulence factors (V. Index = 1.0) (Table S1).

The virulence index mean values differed between isolates from sloths belonging to different animal status groups: ‘hand-reared’‒V. index mean value = 0.35, ‘rehabilitated and released‒V. index mean value = 0.49, and ‘wild’‒V. index mean value = 0.43 (Fig. 2B). A statistically significant difference between V. Index mean values of isolates from rehabilitated and released sloths (‘rehabilitated and released’ group) and the values of isolates from sloths being hand-reared (‘hand-reared’ group) was found (*p* = 0.012; *p*_adj_ = 0.016) (Fig. 2B; Table 3). A significant difference was also observed for the isolates’ virulence index mean values between animals that were in captivity for a period less than or equal to one year, and animals. rehabilitated for more than one year, being higher for isolates from sloths from the latter group (*p* = 0.013; *p*_adj_ = 0.023) (Table 3). No significant differences were detected from the isolates’ virulence indices between male and female sloths (Table 3).

## Discussion

This study represents, to the best of our knowledge, the first description of the isolation of antimicrobial-resistant *Enterobacterales* from two-finger (*Choloepus hoffmanni*) and three-finger sloths (*Bradypus variegatus*) of Costa Rica. This study also provides an insight into the influence of sloths’ rehabilitation on the antimicrobial resistance and virulence profiles of different *Enterobacterales* species.

Oral and rectal swab samples constituted quick, effective, and non-traumatic techniques for the isolation and posterior identification of aerobic Gram-negative bacteria of wild and captive sloths. Also, the collection of both oral and rectal samples allowed the isolation of a wider range of *Enterobacterales* species.

The prevalence of bacteria non-susceptible to three or more antimicrobials was 40.0%, with 17.5% of isolates revealing a multidrug resistance profile. One extensively drug-resistant (XDR) *E. coli* isolate was detected, which was isolated from a sloth previously treated with a broad-spectrum antimicrobial (enrofloxacin), due to pneumonia (Table S2).

All isolates were susceptible to gentamicin, which is in line with previous studies performed on wildlife resistant bacteria (Grassotti et al., 2018). In contrast, the highest levels of resistance were detected for meropenem (55.0%), especially for *E. coli* (59.0% of isolates resistant to meropenem) and *C. youngae* (88.9% of isolates resistant or intermediately resistant to meropenem). This finding raises concern due to the categorization of carbapenems as antimicrobials of ‘last resort’ for the treatment of severe infections (Raza et al., 2020).

The detection of resistance to an antimicrobial not used in sloths (meropenem), could be explained by horizontal gene transfer of resistance determinants from environmental bacteria to commensal bacteria. The co-existence of different ARGs in the same mobile genetic element, *i.e*., plasmids, can result in the transfer of the whole group, and bacteria can acquire a resistant gene, even in the absence of direct selection from an antimicrobial (Stoddard et al., 2009; Da Costa, Loureiro & Matos, 2013).

Studies on carbapenem resistance in bacteria isolated from humans and animals in Costa Rica are scarce. However, a recent study revealed high levels of antimicrobial susceptibility for *E. coli* isolated from fecal samples of Baird’s tapirs (*Tapirus bairdii*) from Costa Rica, which results contrast from the ones of this study (59.0% of meropenem-resistant *E. coli* isolates) (Rojas-Jiménez et al., 2019). Also, in a study performed on house-hold dogs, no isolates revealed a resistance phenotype against carbapenems (Rodríguez-González et al., 2020). On the other hand, high levels of resistance towards this antimicrobial class were previously detected in clinical isolates of *Pseudomonas aeruginosa* (63.1%) from some of the main hospitals in San Jose (Toval et al., 2015). The detection of resistance to carbapenems in isolates obtained from sloths being hand-raised and rehabilitated in a wildlife facility raises concern for public health, due to the possible transmission of these meropenem-resistant bacteria to humans that may directly or indirectly contact with these animals.

In the present study, resistance towards fluoroquinolones, especially enrofloxacin (12.5% of isolates) and cephalosporins, namely cephalexin (27.5%) and ceftazidime (5.0%) were also detected. These findings raise concern due to the medical importance of these antimicrobials (carbapenems (meropenem), fluoroquinolones (enrofloxacin), and third-generation cephalosporins (ceftazidime)) for both human and veterinary medicine, including wildlife (Vittecoq et al., 2016; McEwen & Collignon, 2018; Baros Jorquera et al., 2021). Resistance to cephalosporins, including ceftazidime was recently detected in *E. coli* isolates in healthy household dogs from Costa Rica (Rodríguez-González et al., 2020). Previous research also revealed high levels of AMR towards first generation cephalosporins and enrofloxacin in clinical samples of food-producing animals in Costa Rica (Mayorga et al., 2015; Andino-Molina et al., 2019). According to Andino-Molina et al., 2019, the frequent and extensive uncontrolled use of quinolones and cephalosporins is a common practice in Central American husbandry.

Regarding the MAR index results, *C. youngae* (MAR index mean value = 0.24) and *E. coli* (MAR index mean value = 0.21) isolates revealed the highest MAR index values. These bacterial species (*C. youngae* and *E. coli*) were exclusively isolated from animals with previous human exposure (‘hand-reared’ and ‘rehabilitated and released’ groups). The antimicrobials mostly used for the treatment of rehabilitated sloths are enrofloxacin and amikacin and the approximate length of treatment is 3 weeks. In this study, there was no association between antimicrobial treatment and the isolates’ MAR index values, which is in line with the study performed by Baros Jorquera et al. (2021). However, this association was observed for isolates 1.01, 1.02, and 2.07, which were resistant to enrofloxacin and obtained from sloths previously treated with this antimicrobial. Furthermore, although not statistically significant, the levels of resistance in bacteria isolated from samples of animals rehabilitated for long periods and treated with large-spectrum antibiotics (‘rehabilitated and released’ group) were higher compared to the ones of isolates from samples of other animal status groups (‘hand-reared’ and ‘wild’).

Previous studies support that time in captivity and antibiotic treatment can have a large influence on the antimicrobial resistance outcome of wildlife bacteria (Stoddard et al., 2009; Baros Jorquera et al., 2021), including a study performed on a wildlife rehabilitation center from Latin America (Baros Jorquera et al., 2021). How the sloths under study acquired ARB is unknown. The dissemination of ARB could have occurred *via* staff and wildlife professionals, fomites, or through water and food supply (Stoddard et al., 2009). Also, sloths could acquire ARB through environmental exposure to antimicrobial residues, antimicrobial resistant bacteria, or antimicrobial resistant genes. In fact, due to the increasing anthropogenic interference of sloths’ habitats in Costa Rica, these animals are at a higher risk to contact with anthropized environments, including farms and urban areas, which can present various sources of AMR. Lastly, we should also consider that bacteria might be intrinsically resistant to antimicrobials, independently of antimicrobial selective pressures (Fletcher, 2015).

Sloths rehabilitated and released back into the wild may potentially introduce antimicrobial-resistant strains into new ecological niches and transmit them to wild individuals, contributing to AMR dissemination. Multidrug-resistant isolates were isolated from samples collected from sloths both two months and two years after release, which raises concern on the extent of the impact of sloths as AMR reservoirs. In humans, it is suggested that a short course of an antimicrobial, can cause ARB to persist in the feces for 3 months (Cunha, 2000). Sloths’ fecal droppings containing ARB could contaminate soils and rivers which could result in the transfer of resistant bacteria or genes to other wildlife species and even livestock. In fact, although the Release Site is in a region (Sarapiqui) mostly characterized by a wild environment, the level of human activity and livestock surged in the past years, which could result in an indirect association between the released sloths and both farm and domestic animals. Therefore, released sloths could act as efficient AMR reservoirs and epidemiological links between humans, livestock, and natural environments, contributing to the dissemination of AMR.

Regarding virulence characterization, the isolates were able to produce several virulence factors, including lipases, hemolysins, and biofilms. Virulence factors give bacteria an increased capability to colonize and infect animal hosts, especially when immunocompromised (Da Costa, Loureiro & Matos, 2013).

*E. aerogenes* isolate revealed the highest virulence index. In fact, although *E. cloacae* is currently more frequently isolated in clinical settings, *E. aerogenes* usually reveals higher virulence ability (Davin-Regli & Lavigne, 2019). *E. amnigenus* also revealed high virulence indices. For opportunistic pathogens, as *Enterobacter* sp., the expression of virulence factors implicated in cell adherence and invasion (*e.g*., lipases and proteases) can be crucial for tissue colonization and subsequent infection (Mishra, Patole & Mohapatra, 2017), which raises concerns about the pathogenic potential of these *Enterobacter* species.

One *E. coli* isolate revealed the ability to produce all tested virulence factors (V. Index = 1.0), also revealing a multidrug-resistance profile. This isolate was obtained from a sample collected from a two-finger sloth rehabilitated for approximately one year and previously treated with a broad-spectrum antibiotic (amoxicillin-clavulanate), due to a deep dermal abscess in the neck (Table S2).

A statistically significant difference between the V. Index values of isolates from sloths rehabilitated and released (‘rehabilitated and released’ group) and the V. index values of isolates from animals being hand-reared (‘hand-reared’ group) was found. The lack of a significant difference between the V. Index values of isolates from samples of ‘rehabilitated and released’ sloths and those from ‘wild’ animals may be explained by the low number of isolates obtained for the ‘wild’ group. Bacteria develop virulence mainly through the acquisition of virulence genes encoded on mobile pathogenicity islands or segments of the genome associated with mobility elements (Bliven & Maurelli, 2016). The horizontal gene transfer of these mobile DNA elements allows the rapid spread of advantageous alleles within host-associated bacteria or through the exchange of microorganisms between different host groups (*e.g*., humans and wild animals) (Bliven & Maurelli, 2016). The high virulence indices observed for isolates from ‘rehabilitated and released’ sloths highlight the potential for virulent bacteria and/or virulence mechanisms to be acquired during rehabilitation and be unintentionally disseminated to both wild sloths’ and other animals’ species, as well as their respective habitats.

*Enterobacterales* species identified in this study, including *E. coli*, *E. cloacae*, and *Salmonella* spp., were previously associated with sloths’ bacterial diseases, especially pneumonia and enteritis (Dünner & Pastor, 2017). The detection of potentially pathogenic bacterial species revealing high levels of antimicrobial resistance and complex virulence profiles raises concern on the impact of such bacteria on sloths’ health and rehabilitation. Moreover, the identification of resistance towards antibiotics commonly used in wildlife facilities of Costa Rica, such as amoxicillin/clavulanate, enrofloxacin, and tetracycline, may compromise the ability to successfully treat disease, which is detrimental to sloths’ and Costa Rican wildlife rehabilitation programs. Nevertheless, the presence of the isolated bacterial species in samples collected from sloths is not mandatorily associated with disease, and there is still a knowledge gap on the risk of ARB, especially those with a virulent profile, on the health of these animals (Grilo et al., 2020).

*Shigella* sp. was isolated from a sample of the oral cavity of a sloth. Although humans are the conventional hosts of *Shigella* sp., the expansion of *Shigella*’s hosts to other animal species has been recently reported (Zhu et al., 2018). Although the *Shigella* sp. isolate in this study only revealed resistance to ampicillin, the presence of this serious gastrointestinal pathogen in a sloth’s oral cavity should not be disregarded. *Salmonella* spp. was also isolated and identified in the present study. Wild sloths carrying *Salmonella* species pose a significant risk to human health, particularly to wildlife professionals, as *Salmonella* infections in humans have been associated with direct contact with wild animals (Thomas et al., 2017).

The levels of antimicrobial resistance and virulence and the zoonotic potential of the *Enterobacterales* species isolated in the present study may represent a significant risk to human and animal health, underlining the requirement for an integrative One Health approach in the surveillance of pathogens among humans and wildlife populations, including sloths. The solutions should not focus on stopping wildlife rehabilitation programs, but to improve antimicrobial resistance monitoring in wildlife facilities, and to make wildlife professionals aware of the importance of implementation and management of antimicrobial treatment based on bacterial culture and antimicrobial susceptibility testing.

The limitations of this study include the potentially biased selection of species on culture-based techniques, the lower sensitivity of the disk diffusion method and the API identification system, compared with molecular methods, such as whole genomic analysis and metagenomic analysis.

Also, the small number of isolates obtained from samples collected from wild and three-finger sloths (*B. variegatus*), did not allow the analysis of the potential differences between sloths’ species and the impact of wild populations. Despite these limitations, this work provides valuable data, by assessing the antimicrobial resistance issue on two sloths’ species in which was never addressed.

## Conclusions

This study aimed to evaluate the presence of ARB in samples collected from two-finger (*Choloepus hoffmanni*) and three-finger sloths (*Bradypus variegatus*) of Costa Rica. We also aimed to analyze the influence of the rehabilitation of sloths in Costa Rica on the AMR and virulence profiles of sloths’ *Enterobacterales*. Our results showed that wild two-finger and three-finger sloths can carry antimicrobial-resistant bacteria of major importance for veterinary and human medicine, which contributes to the understanding of the AMR issue in wildlife. Moreover, our results revealed that sloths released in their natural habitats after rehabilitation in wildlife facilities may harbor bacteria with higher levels of AMR and virulence. These findings suggest that proximity between humans and wildlife may select for AMR and virulence in wildlife bacteria. Additionally, the bacterial species identified in this study may represent a significant threat to human and animal health, both due to their zoonotic potential and multidrug-resistance and virulence profiles.

Further studies are encouraged aiming at including whole genome sequencing analysis to identify the genetic determinants associated with AMR and virulence, especially for the isolates revealing multidrug resistance profiles and resistance to ‘last resort’ antimicrobials, such as meropenem. It would be equally relevant to assess AMR and virulence in bacteria from the environment where sloths are rehabilitated and people working in close contact with these animals. Finally, it would be relevant to further evaluate the role of released sloths as dispersers of AMR and the potential transmission of antimicrobial resistance and virulence among wild populations.

## Supplemental Information

10.7717/peerj.12911/supp-1 Supplemental Information 1 Isolates’ antimicrobial resistance and virulence profiles. Number (n°.), rectal swab sample (R), oral swab sample (O), ampicillin (AMP), amikacin (AK), ciprofloxacin (CIP), enrofloxacin (ENR), imipenem (IMP), meropenem (MEM), cephalexin (CL), ceftazidime (CAZ), chloramphenicol (C), tetracycline (T), hemolysins (HEM), gelatinases (GEL), lecithinases (LEC), proteases (PT), lipases (LIP), biofilm (BF), beta-haemolysis (β), gamma-haemolysis (γ), multiple antibiotic resistance index (MAR index), virulence index (V. Index), positive (+), negative (-), multidrug-resistant isolates (*), extensively drug-resistant isolate (^†^). The animal number refers to Table S2 (“Animal data. Detailed data of the sloths under study”).

10.7717/peerj.12911/supp-2 Supplemental Information 2 Animal data. Detailed data of the sloths under study. *Choloepus hoffmanni* (*C. hoffmanni*), *Bradypus variegatus* (*B. variegatus*). The details of the corresponding isolates are presented in Table S1 (“Isolates’ antimicrobial resistance and virulence profiles.”). *The age is approximated.

10.7717/peerj.12911/supp-3 Supplemental Information 3 Area of study. Costa Rica (Lat: 9°37′8.6″N; Long: −84°15′15.0″W) is highlighted in blue. Sampling points: Toucan Rescue Ranch Headquarters–San José (**○**) (Lat: 10°57′6.2″N; Long.: −85°08′9.9″W) and Release Site–Sarapiqui (*) (Lat: 10°28′24.682″N; Long: −84°1′0.2712″W). Map created using R package “rnaturalearth” v0.1.0 (South, 2017) and “ggplot2” v3.3.3 (Wickham, 2016).

## Additional Information and Declarations

### Data Availability

The following information was supplied regarding data availability: The raw data is available in the Supplemental Files.

## References

[ref-1] Alduina R, Gambino D, Presentato A, Gentile A, Sucato A, Savoca D, Filippello S, Visconti G, Caracappa G, Vicari D, Arculeo M (2020). Is *Caretta caretta* a carrier of antibiotic resistance in the mediterranean sea?. Antibiotics.

[ref-2] Andino-Molina M, Barquero-Calvo E, Seyboldt C, Schmoock G, Neubauer H, Tzoc E, Rodríguez C, Quesada-Gómez C (2019). Multidrug-resistant *Clostridium difficile* ribotypes 078 and 014/5-FLI01 in piglets from Costa Rica. Anaerobe.

[ref-3] Baros Jorquera C, Moreno-Switt AI, Sallaberry-Pincheira N, Munita JM, Flores Navarro C, Tardone R, González-Rocha G, Singer RS, Bueno I (2021). Antimicrobial resistance in wildlife and in the built environment in a wildlife rehabilitation center. One Health.

[ref-4] Bates D, Maechler M, Bolker B, Walker S (2015). Fitting linear mixed-effects models using lme4. Journal of Statistical Software.

[ref-5] Bliven KA, Maurelli AT (2016). Evolution of bacterial pathogens within the human host. Virulence Mechanisms of Bacterial Pathogens.

[ref-6] Chrisope GL, Fox CW, Marshall RT (1976). Lecithin agar for detection of microbial. Applied and Environmental Microbiology.

[ref-8] CLSI (2020). Performance standards for antimicrobial disk and dilution susceptibility tests for bacteria isolated from animals.

[ref-9] Cunha BA (2000). Antibiotic resistance. Medical Clinics of North America.

[ref-10] Da Costa PM, Loureiro L, Matos AJF (2013). Transfer of multidrug-resistant bacteria between intermingled ecological niches: the interface between humans, animals and the environment. International Journal of Environmental Research and Public Health.

[ref-11] Davin-Regli A, Lavigne J (2019). *Enterobacter* spp.: update on taxonomy, clinical aspects, and emerging antimicrobial resistance. Clinical Microbiology Reviews.

[ref-12] Dill-Mcfarland KA, Weimer PJ, Pauli JN, Peery MZ, Suen G (2016). Diet specialization selects for an unusual and simplified gut microbiota in two- and three-toed sloths. Environmental Microbiology.

[ref-13] Dünner CO, Pastor GN (2017). Manual de manejo, medicina y rehabilitación de perezosos. https://www.xenarthrans.org/wp-content/uploads/2019/10/32Manual-de-manejo-medicina-y-rehabilitacio%CC%81n-de-perezosos_Dunner-y-Pastor-2017.pdf.

[ref-14] Elder BL, Trujillo I, Blazevic DJ (1977). Rapid deoxyribonuclease test with methyl green. Journal of Clinical Microbiology.

[ref-15] Fernandes M, Grilo ML, Carneiro C, Cunha E, Tavares L, Patino-Martinez J, Oliveira M (2021). Antibiotic resistance and virulence profiles of gram-negative bacteria isolated from loggerhead sea turtles (*Caretta caretta*) of the Island of Maio, Cape Verde. Antibiotics.

[ref-16] Fletcher S (2015). Understanding the contribution of environmental factors in the spread of antimicrobial resistance. Environmental Health and Preventive Medicine.

[ref-17] Freeman DJ, Falkiner FR, Keane CT (1989). New method for detecting slime production by coagulase negative staphylococci. Journal of Clinical Pathology.

[ref-18] Grassotti TT, De Angelis Zvoboda D, Da Fontoura Xavier Costa L, De Araújo AJG, Pereira RI, Soares RO, Wagner PGC, Frazzon J, Frazzon APG (2018). Antimicrobial resistance profiles in *Enterococcus* spp. Isolates from fecal samples of wild and captive black capuchin Monkeys (*Sapajus nigritus*) in South Brazil. Frontiers in Microbiology.

[ref-19] Grilo ML, Sousa-santos C, Robalo J, Oliveira M (2020). The potential of *Aeromonas* spp. from wildlife as antimicrobial resistance indicators in aquatic environments. Ecological Indicators.

[ref-20] Holm S (1979). Board of the foundation of the Scandinavian journal of statistics a simple sequentially rejective multiple test procedure a simple sequentially rejective multiple test procedure. Source: Scandinavian Journal of Statistics.

[ref-21] Johnson DI (2017). Bacterial pathogens and their virulence factor.

[ref-22] Krumperman PH (1983). Multiple antibiotic resistance indexing of *Escherichia coli* to identify high-risk sources of faecal contamination of water. Environmental Science and Pollution Research.

[ref-23] Magiorakos AP, Srinivasan A, Carey RB, Carmeli Y, Falagas ME, Giske CG, Harbarth S, Hindler JF, Kahlmeter G, Olsson-Liljequist B, Paterson DL, Rice LB, Stelling J, Struelens MJ, Vatopoulos A, Weber JT, Monnet DL (2012). Multidrug-resistant, extensively drug-resistant and pandrug-resistant bacteria: an international expert proposal for interim standard definitions for acquired resistance. Clinical Microbiology and Infection.

[ref-24] Mayorga M, Rodríguez-Cavallini E, López-Ureña D, Barquero-Calvo E, Quesada-Gómez C (2015). Identification and antimicrobial susceptibility of obligate anaerobic bacteria from clinical samples of animal origin. Anaerobe.

[ref-25] McEwen SA, Collignon PJ (2018). Antimicrobial resistance: a one health perspective. Microbiology Spectrum.

[ref-26] Midorikawa Y, Nakamura S, Phetsouvanh R, Midorikawa K (2014). Detection of non-typhoidal salmonella using a mechanism for controlling hydrogen sulfide production. Open Journal of Medical Microbiology.

[ref-27] Mishra M, Patole S, Mohapatra H (2017). Draft genome sequences of nonclinical and clinical *Enterobacter cloacae* isolates. Genome Announcements.

[ref-28] Moraes-Barros N, Chiarello A, Plese T (2014). *Bradypus variegatus*. The IUCN Red List of Threatened Species 2014: e.T3038A47437046. https://www.iucnredlist.org/species/3038/47437046.

[ref-29] Pavlickova S, Klancnik A, Dolezalova M, Mozina SS, Holko I (2017). Antibiotic resistance, virulence factors and biofilm formation ability in *Escherichia coli* strains isolated from chicken meat and wildlife in the Czech Republic. Journal of Environmental Science and Health, Part B.

[ref-30] Plese T, Chiarello A (2014). *Choloepus hoffmanni*. The IUCN Red List of Threatened Species 2014: e.T4778A47439751. https://www.iucnredlist.org/species/4778/47439751.

[ref-31] Power ML, Emery S, Gillings MR (2013). Into the wild: dissemination of antibiotic resistance determinants via a species recovery program. PLOS ONE.

[ref-32] Powers EM, Latt TG (1977). Simplified 48-hour IMVic test. Microbiology.

[ref-33] Radhouani H, Silva N, Poeta P, Torres C, Correia S, Igrejas G (2014). Potential impact of antimicrobial resistance in wildlife, environment, and human health. Frontiers in Microbiology.

[ref-34] Raza A, Ngieng SC, Sime FB, Cabot PJ, Roberts JA, Popat A, Kumeria T, Falconer JR (2020). Oral meropenem for superbugs: challenges and opportunities. Drug Discovery Today.

[ref-35] Rodríguez-González MJ, Jiménez-Pearson MA, Duarte F, Poklepovich T, Campos J, Araya-Sánchez LN, Chirino-Trejo M, Barquero-Calvo E (2020). Multidrug-resistant CTX-M and CMY-2 producing *Escherichia coli* isolated from healthy household dogs from the great metropolitan area, Costa Rica. Microbial Drug Resistance.

[ref-36] Rojas-Jiménez J, Brenes-Mora E, Alcázar-García P, Arguedas-Porras R, Barquero-Calvo E (2019). Pansusceptible *Escherichia coli* isolates obtained from faeces of free-ranging Baird’s tapirs (*Tapirus bairdii*) suggests a low selective pressure for resistance determinants in the northwestern region of the Talamanca Mountain Range, Costa Rica. Journal of Global Antimicrobial Resistance.

[ref-37] Sacristán I, Esperón F, Acuña F, Aguilar E, García S, López MJ, Cevidanes A, Neves E, Cabello J, Hidalgo-Hermoso E, Poulin E, Millán J, Napolitano C (2020). Antibiotic resistance genes as landscape anthropization indicators: using a wild felid as sentinel in Chile. Science of the Total Environment.

[ref-38] Semedo-Lemsaddek T, Nóbrega CS, Ribeiro T, Pedroso NM, Sales-Luís T, Lemsaddek A, Tenreiro R, Tavares L, Vilela CL, Oliveira M (2013). Virulence traits and antibiotic resistance among enterococci isolated from Eurasian otter (*Lutra lutra*). Veterinary Microbiology.

[ref-39] Singh SK, Ekka R, Mishra M, Mohapatra H (2017). Association study of multiple antibiotic resistance and virulence: a strategy to assess the extent of risk posed by bacterial population in aquatic environment. Environmental Monitoring and Assessment.

[ref-40] Sokol PA, Ohman DE, Iglewski BH (1979). A more sensitive plate assay for detection of protease production by *Pseudomonas aeruginosa*. Journal of Clinical Microbiology.

[ref-41] South A (2017). rnaturalearth: world map data from natural earth. https://CRAN.R-project.org/package=rnaturalearth.

[ref-42] Stoddard RA, Atwill ER, Conrad PA, Byrne BA, Jang S, Lawrence J, McCowan B, Gulland FMD (2009). The effect of rehabilitation of northern elephant seals (*Mirounga angustirostris*) on antimicrobial resistance of commensal *Escherichia coli*. Veterinary Microbiology.

[ref-43] Thomas M, Fenske GJ, Antony L, Ghimire S, Welsh R, Ramachandran A, Scaria J (2017). Whole genome sequencing-based detection of antimicrobial resistance and virulence in non-typhoidal *Salmonella enterica* isolated from wildlife. Gut Pathogens.

[ref-44] Toval F, Guzmán-Marte A, Madriz V, Somogyi T, Rodríguez C, García F (2015). Predominance of carbapenem-resistant *Pseudomonas aeruginosa* isolates carrying bla IMP and bla VIM metallo-β-lactamases in a major hospital in Costa Rica. Journal of Medical Microbiology.

[ref-45] Visser IJR, Jaisly F, Mossel DAA (1985). The effect of properties of dried preparations of sulphide iron motility (SIM) agar on the results of motility readings. Journal of Applied Bacteriology.

[ref-46] Vittecoq M, Godreuil S, Prugnolle F, Durand P, Brazier L, Renaud N, Arnal A, Aberkane S, Jean-Pierre H, Gauthier-Clerc M, Thomas F, Renaud F (2016). Antimicrobial resistance in wildlife. Journal of Applied Ecology.

[ref-47] Wickham H (2016). ggplot2: elegant graphics for data analysis.

[ref-48] Zhu Z, Shi Y, Zhou X, Li B, Zhang J (2018). Molecular characterization of fluoroquinolone and/or cephalosporin resistance in *Shigella sonnei* isolates from yaks. BMC Veterinary Research.

