# Supplementary figures and images for: Antimicrobial resistance and virulence profiles of Enterobacterales isolated from two-finger and three-finger sloths (Choloepus hoffmanni and Bradypus variegatus) of Costa Rica
Source: PeerJ. 2022 Mar 11;10:e12911. doi: 10.7717/peerj.12911 (PMC8919844; doi:10.7717/peerj.12911)

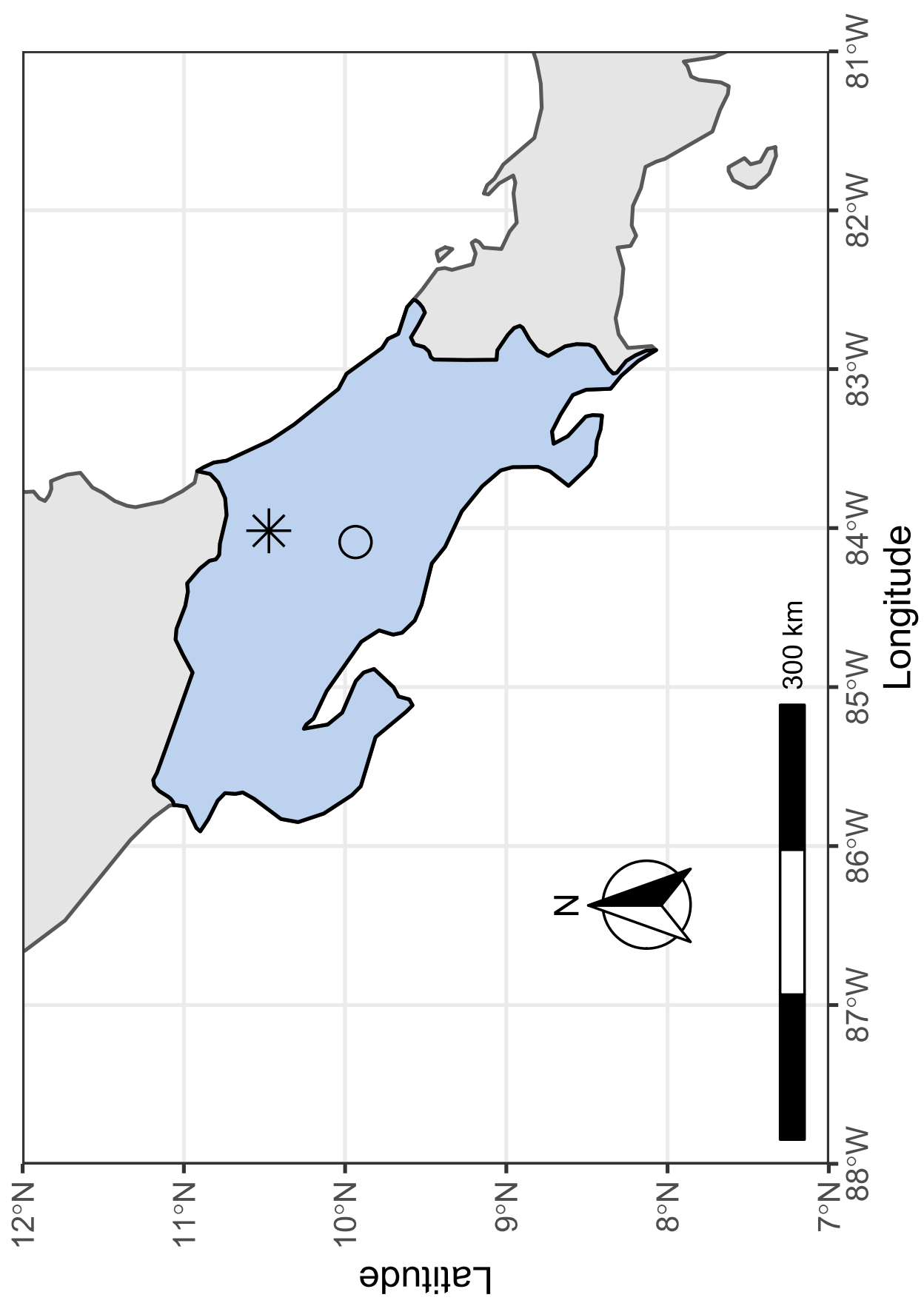

Supplement: Supplemental Information 3 — Costa Rica (Lat: 9°37′8.6″N; Long: −84°15′15.0″W) is highlighted in blue. Sampling points: Toucan Rescue Ranch Headquarters–San José (○) (Lat: 10°57′6.2″N; Long.: −85°08′9.9″W) and Release Site–Sarapiqui (*) (Lat: 10°28′24.682″N; Long: −84°1′0.2712″W). Map created using R package “rnaturalearth” v0.1.0 (South, 2017) and “ggplot2” v3.3.3 (Wickham, 2016). [file peerj-10-12911-s003.pdf]
